# Radiation-Induced Senescence Reprograms Secretory and Metabolic Pathways in Colon Cancer HCT-116 Cells

**DOI:** 10.3390/ijms22094835

**Published:** 2021-05-03

**Authors:** Chandrasekharam N. Nagineni, Sarwat Naz, Rajani Choudhuri, Gadisetti V. R. Chandramouli, Murali C. Krishna, Jeffrey R. Brender, John A. Cook, James B. Mitchell

**Affiliations:** 1 Radiation Biology Branch, Center for Cancer Research, National Cancer Institute, National Institutes of Health, Bethesda, MD 20892, USA; sarwat.naz@nih.gov (S.N.); rajani.choudhuri@nih.gov (R.C.); murali@helix.nih.gov (M.C.K.); jeffrey.brender@nih.gov (J.R.B.); john.cook@nih.gov (J.A.C.); jbm@helix.nih.gov (J.B.M.); 2 Genepria Consulting Inc., Columbia, MD 21046, USA; mouli@genepria.com

**Keywords:** senescence, colon cancer, HCT-116, ionizing radiation, secretome, metabolic pathways, glycolysis, chemokines, cytokines

## Abstract

Understanding the global metabolic changes during the senescence of tumor cells can have implications for developing effective anti-cancer treatment strategies. Ionizing radiation (IR) was used to induce senescence in a human colon cancer cell line HCT-116 to examine secretome and metabolome profiles. Control proliferating and senescent cancer cells (SCC) exhibited distinct morphological differences and expression of senescent markers. Enhanced secretion of pro-inflammatory chemokines and IL-1, anti-inflammatory IL-27, and TGF-β1 was observed in SCC. Significantly reduced levels of VEGF-A indicated anti-angiogenic activities of SCC. Elevated levels of tissue inhibitors of matrix metalloproteinases from SCC support the maintenance of the extracellular matrix. Adenylate and guanylate energy charge levels and redox components NAD and NADP and glutathione were maintained at near optimal levels indicating the viability of SCC. Significant accumulation of pyruvate, lactate, and suppression of the TCA cycle in SCC indicated aerobic glycolysis as the predominant energy source for SCC. Levels of several key amino acids decreased significantly, suggesting augmented utilization for protein synthesis and for use as intermediates for energy metabolism in SCC. These observations may provide a better understanding of cellular senescence basic mechanisms in tumor tissues and provide opportunities to improve cancer treatment.

## 1. Introduction

Senescence arising spontaneously or by oncogene-induced carcinogenesis, ionizing radiation (IR) during radiotherapy or by anti-cancer drug treatment is generally considered as a cancer suppressor program in tumor tissues [1,2,3,4,5]. The presence of senescent cancer cells (SCC) has been demonstrated in several solid tumors, premalignant, malignant and in pre- and post-treatment conditions [1,2,3,6,7]. Under certain conditions, the presence of SCC in malignant lesions may prevent the expansion of cancer cells leading to stable disease but without reducing tumor burden [1,2,3,8,9]. SCC within solid tumors exhibits a characteristic pattern of secretion of many factors with the potential to influence cancer suppression or progression. The secretion of molecules by senescent cells, generally different from proliferating cells, is known as senescence-associated secretory phenotype (SASP). In addition to SASP, SCC may also reprogram metabolic pathways to meet altered requirements in the tumor microenvironment [10,11,12,13]. References OK JBM; however, the references are not superscripted.

Senescence plays a critical role in aging and cancer [11,12,14,15,16]. Cellular senescence is an irreversible cell cycle arrest caused by several factors [11,12,14,15,16]. Physiological senescence is generally induced by replicative exhaustion due to telomere shortening and a lack of telomerase activity [17,18]. Exposure of cells to IR induces DNA damage by causing double or single strand breaks due to direct actions on free bases and nucleotides and the production of reactive oxygen species [19,20]. Cellular senescence is also induced by many anti-cancer drugs such as doxorubicin, etoposide and oncogene activation during tumorigenic processes [10,11,12,14,21]. Typical characteristics of senescent cells include inhibition of cellular proliferation and morphological changes with enlarged, flattened shape, intracytoplasmic granules, vacuoles and fragmented nucleoli [12,14,15,16,22,23]. The expression of many molecular markers such as p16INK4a, p15INK4b, p53, p21 are induced or inhibited during senescence [11,12,14,15,16,22,23]. In addition, the expression of ƴ-H2AX (DNA damage marker), senescence-associated heterochromatin foci (SAHFS), and histone H3 lysine 9 trimethylation are associated with senescence due to chromatin modification and damage [9,22,23].

Radiation therapy is part of the cancer treatment regimen for many solid tumors either alone or in combination with surgery or chemotherapy. IR treatment is given as external beam radiation or as intraoperative radiation for many solid tumors such as colorectal, breast, pulmonary, prostate, head and neck, and other cancers [24,25,26,27,28]. Colorectal cancers are the second most common tumors in men and women [29,30,31], and radiotherapy has been shown to be beneficial in treating these patients [24,25,28]. IR used for radiotherapy induces DNA damage [19,20,21] and interferes with tumor growth by inhibiting cell proliferation and promoting cell killing by necrosis and apoptosis. Some tumor cells escape cell death and undergo permanent cell cycle arrest, a phenomenon known as senescence [11,12,14,15,16]. The effects of IR on colorectal cancer cell senescence and the consequent changes in metabolic and secretory pathways are largely unknown.

It is well known that proliferating cancer cells reprogram metabolic and secretory pathways to suit the demands of growth and other requirements under altered conditions within the tumor microenvironment [32,33,34]. A limited number of studies used human normal fibroblasts and cancer cell lines to understand the molecular and pathophysiological mechanisms by analyzing intracellular metabolites and the secretion of proteins by cells undergoing senescence in the presence of a variety of agents [12,23,35,36,37].

The presence of senescent cells and proliferating cancer cells within tumor tissues has been reported [6,7,9,29,30,38]. Senescent cells within the solid tumors exhibit a pattern of secretion of many factors (SASP) distinct from proliferating neighboring cells within the tumor microenvironment. SASP consists of a variety of protein molecules that can influence leucocyte infiltration, inhibition or proliferation of cells, extracellular matrix integrity and initiation of metastasis from the tumor. Because of the complexity of tumor tissue, it is difficult to distinguish the origin of SASP from senescent or proliferating cells within the tumor tissue due to diffusion, binding to neighboring cells and matrix as well as many interacting factors. Therefore, we used in vitro cell culture models to examine the metabolome and secretome of proliferating and IR-induced senescent HCT-116 cells. HCT-116, a human colon cancer cell line, is one of the best characterized cells representing colon cancer and was widely used by many investigators to address the mechanisms of oncogenic senescence, autophagy and other cell death as well as effects of various synthetic drugs and natural products for anticancer activities [39,40,41]. However, there no studies addressing the effects of radiation (X-rays) on HCT-116 or other colon cancer cells, even though radiation therapy is part of colon cancer treatment. Therefore, it is important to initiate studies to address the various aspects of the effects of ionizing radiation on HCT-116 cells in order to delineate the underlying pathophysiological processes. 

The aim of the current study was to investigate changes in the steady state kinetics of different metabolites in IR-induced senescence of HCT-116 colon cancer cells, by using capillary electrophoresis mass spectrometry (CE-MS)-based metabolomics. Further, multiplex luminescence assay technologies were used to identify secreted proteins. We report here that compared to proliferating HCT-116 cells, senescent HCT-116 cells undergo reprogramming of metabolic and secretory pathways as they enter into the non-proliferating senescence state. 

## 2. Results

### 2.1. HCT-116 Cells Exhibit Cellular Senescence Phenotype Following Ionizing Radiation 

Senescence-associated beta-galactosidase (SA-βgal) expression has been the standard method for detecting senescent cells both in tissue sections and in cultured cells [39]. HCT-116 (human colon cancer cell line) and SCCVII (murine oral squamous carcinoma cells) were exposed to IR in vitro. Cellular morphology and SA-βgal staining following exposure to IR was used to detect the extent of the senescent phenotype (Figure S1). HCT-116 cells exhibited significant characteristics of typical senescence, namely enlarged cell size, intracellular granules and intense positive staining for SA- βgal compared to SCCVII cells. Thus, we used human HCT-116 cells as a model system to understand the reprogramming of metabolic and secretory pathways in senescent cells in response to IR-induced stress. 

### 2.2. IR Dose and Time Dependent Senescence Responses by HCT-116 Cells 

We conducted a number of pilot studies to identify the cell plating density, IR dose and time course of expression of senescence characteristic markers. Senescent cells typically have enlarged cell size, with many vacuoles and fragmented nuclei compared to non-irradiated proliferating HCT-116 cells. After conducting preliminary studies, we were able to identify senescent cells by observing live cells under inverted microscope. Fixed cells were used for measuring the dimensional area of senescent cells and were found to be 5–8 times more than proliferating cells (data not shown). The enlarged cell size, viability and senescence (SA-βgal staining) were observed even after 7 days post-IR (Figure S2). These results indicate that senescent HCT-116 cells are alive and may be under physiological state of stress. 

**Figure 1:**
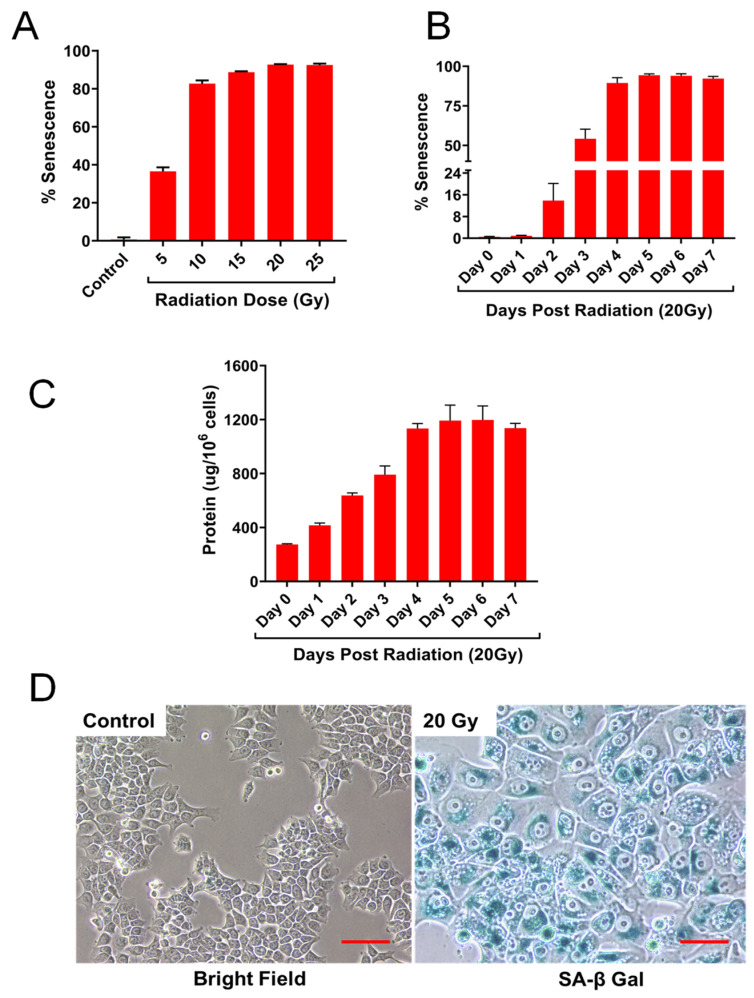
Ionizing radiation induces senescence in human colon cancer cell line, HCT-116. (**A**) Effect of IR dose on senescence in HCT-116 cells. (**B**) Time course of senescence induction in HCT-116 cells after 20 Gy IR treatment. (**C**) Levels of cellular protein at various post-IR days in HCT-116 cells exposed to 20 Gy. Control cells were used on the day of IR but without IR. Results are from one typical experiment with triplicate samples representing at least two other experiments. (**D**) Representative photomicrographs of HCT-116 cells. Cells were fixed and stained for SA-βgal. Red bar represents 100 µM.

HCT-116 cells (proliferating control) were exposed to various doses of IR, 5 to 25 Gy and cells were fixed and stained for SA-βgal after 5 days post-IR. The presence of senescent cells was less than 2% in control non-irradiated cultures. At 5 Gy, approximately 30% exhibited positive staining for senescence with a progressive increase in senescent cells with increasing doses of IR (Figure 1A). At 20 Gy, typically 90% ± 5% of the cells showed the presence of senescent cells and no proliferating cells were detected. Next, we examined the time course of senescent cell appearance as a function of time post-IR days.

(Figure 1B). Two days post-IR, less than 15% cells exhibited senescence followed by approximately 50% at day 3 post-IR. There were no substantial differences in senescence from 4 to 7 days post-IR. After IR, as expected, cell cycle progression was blocked, and non-senescent cells probably were eliminated by apoptosis, autophagy and other cell death phenomena. Therefore, only viable senescent cells remained attached to the dish (Figure S3). Next, we examined the levels of total protein by following the number of adherent cells at various times post-IR. The amount of protein (per cell basis) increased significantly with increasing time post-IR. At 4 to 6 days post-IR, protein levels increased 4- to 6-fold higher than proliferating control cells (Figure 1C). Based on these results, cells were exposed to 20 Gy and analysis for metabolomics and secretome studies were done post-IR day 4 or 5 to obtain the maximum number of senescent cells for analysis as shown in Figure 1D. 

### 2.3. Detection of Senescence-Associated Proteins, P53 and p21 by Immunoblot Analysis

Protein extracts prepared from control (non-IR) and senescent cells at various days post-IR or as a function of various IR doses were used for analysis. Both p53 and p21 proteins, which were barely detectable in control cells, increased significantly in IR-induced senescent cells. The effect of IR doses of 5, 10, 15 and 20 Gy on the expression of p53 and p21 four days post-IR was examined. Even at 5 Gy IR, a clear increase in p53 and p21 protein was observed compared to control cells (Figure S4). 

### 2.4. Secretion of Cytokines and Chemokines by Senescent HCT-116 (SCC) Cell Cultures

**Figure 2:**
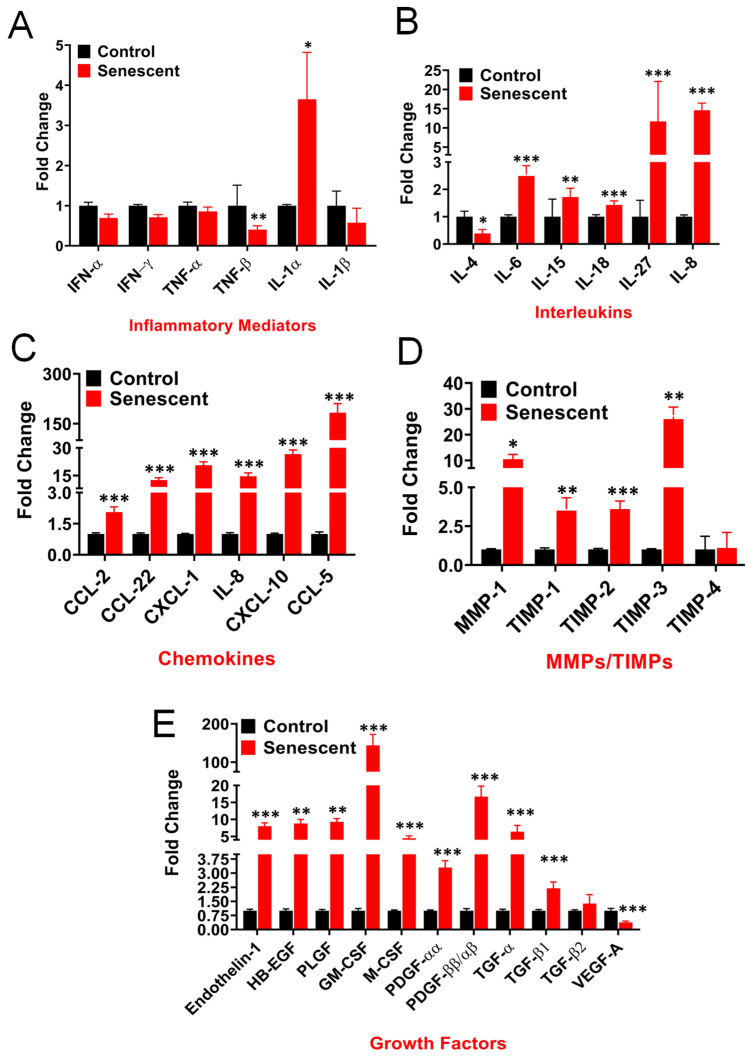
Proteins secreted by control and senescent HCT-116 cells (SCC). Data shown in the figures are expressed as fold changes in senescent cells relative to control and are means ± SD of four individual samples. (**A**) The secretion of inflammatory mediators by control and SSC. (**B**) The secretion of interleukins by control and SCC. No detectable secretion of the following cytokines was observed in both control and SCC: IL-2, IL-3, IL-5, IL-7, IL-9, IL-10, IL-12, IL-13, IL-17 and IL-22. (**C**) The secretion of chemokines by control and SCC. No detectable secretion of the following chemokines was observed in both control and SCC: MIP-1α (CCL-3), MIP-1 β (CCL-4), MCP-3 (CCL-7), Eotaxin (CCL-11), MIG (CXCL-9) and Fractalkine (CX3CL-1). (**D**) The secretion of matrix metalloproteases and TIMPs by control and SCC HCT-116. No detectable secretion of the following MMPs was observed in both control and SCC: MMP-2, 3, 7, 8, 9, 10, 12 and 13 (**E**) The secretion of growth factors by control and SCC. No detectable secretion of the following growth factors was observed in both control and SCC: angiopoetin-2, BMP-9, EGF, endoglin, FGF-1, FGF-2, Follistatin, G-CSF, HGF, Leptin, VEGF-C, VEGF-D and TGF-β3. * *p* value < 0.05; ** *p* value < 0.01, *** *p* value < 0.001.

Proinflammatory cytokines, IL-1α, IL-6, IL-8, and IL-27 were significantly secreted by SCC (3.6-fold, *p* < 0.05, 2.5-fold, *p* < 0.001, 14.6-fold, *p* < 0.001, and 11-fold, *p* < 0.001, respectively). Other proinflammatory cytokines, IL-15 and IL-18, were elevated but less than 2-fold (Figure 2A,B and Table S1). All other interleukins, interferons and TNF-α were secreted in very small quantities or at non-detectable levels (Figure 2A,B; Table S1). The secretion of chemokines, chemo-attractants for inflammatory cells, by SCC was significantly increased in the following decreasing order, CCL-5 (RANTES:183 fold), CXCL-10 (IP-10:26 fold), CXCL-1 (GROa:20.6 fold), CXCL-8 (IL:8–14.6 fold), CCL-22 (MDC:12.5 fold) and CCL-2 (MCP-1:2 fold) (Figure 2C; Table S1).

### 2.5. Secretion of Extracellular Matrix Proteins, MMPs and TIMPs, and Angiogenic and Growth Factors by Senescent HCT-116 (SCC) Cell Cultures

Matrix metalloproteases (MMPs) and tissue inhibitors of MMPs (TIMPs) play important roles in regulating extracellular matrix (ECM) turnover and maintenance of structural integrity of ECM. Dissolution of ECM results in loosening of tumor tissue and release of clusters of cancer cells from tumors into circulation and migration to secondary tissue sites for metastasis. TIMP-1, 2 and 3 secretion by SCC were enhanced significantly (3.6-fold, *p* < 0.001, 3.6-fold, *p* < 0.001, and 26-fold, *p* < 0.001, respectively) when compared to proliferating cells (Figure 2D; Table S1). TIMP-4 secretion by SCC was not altered. MMP-1 secretion increased significantly (10.5-fold, *p* < 0.01) but secretion of other MMPs (MMP-2,3,7,8,9,10,12 and 13) was not observed. 

Among angiogenic agents, there was a significant decrease in the secretion of VEGF-A, from 1776 (proliferating) to 675 (SCC) pg/mg protein (0.382 fold, *p* < 0.001) (Figure 2E; Table S1). No detectable secretion of VEGF-C, VEGF-D was noted. Enhanced levels of other angiogenic factors, such as endothelin, PLGF and HB-EGF were also observed in SCC. Secretion of GM-CSF, M-CSF, PDGF-AA and PDGF-AA/AB by SCC increased significantly compared to proliferating cells (Figure 2E; Table S1). In fact, GM-CSF was the most elevated growth factor measured (144-fold, *p* < 0.001). The secretion of both TGF-α and TGF-β1 by SCC increased significantly (6.4-fold, *p* < 0.001 and 2.2-fold, *p* < 0.001, respectively) (Figure 2E; Table S1). 

### 2.6. Analysis of Metabolites in Control and Senescent HCT-116 Cells (SCC) 

Metabolomic profiles of proliferating (control) and SCC were analyzed by c-Scope/CARNIOSCOPE (Human Metabolome Technologies Inc.) that consists of a total 116 metabolites. 

**Figure 3:**
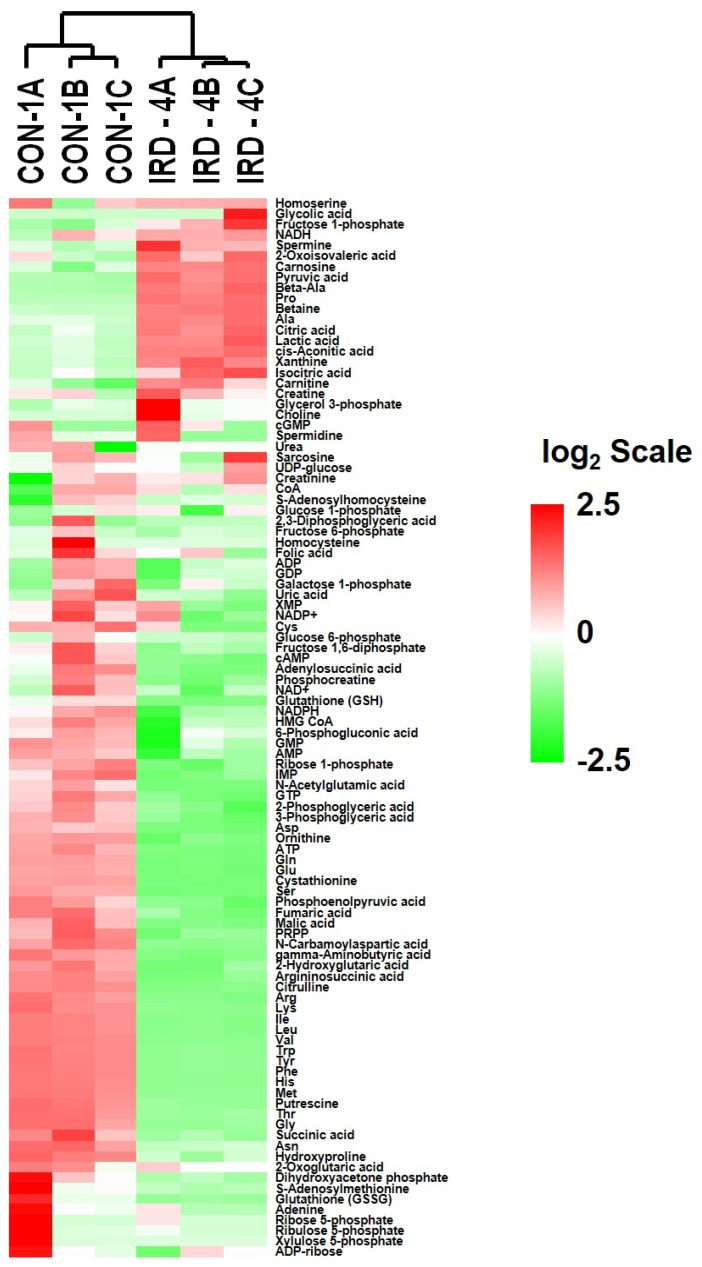
Metabolite expression heat Map related to control and senescent HCT-116 cells. CON-1A, CON-1B and CON-1C are triplicate samples of control (non-IR) while IRD-4A, IRD-4B and IRD-4C are triplicate samples of 20 Gy IR-induced senescent cells. The heat map shown is an agglomerative clustering of 100 metabolites. Mean centered logarithmic values of the abundances and clustering is determined by average linkage algorithm and correlation as distance metric.

The levels of metabolites were expressed as n moles/gram protein in control and senescent cells. Heat maps of the expression of metabolites (Figure 3) indicate a distinct increase in the number of metabolites in senescent cells (IRD-4A, 4B and 4C), while most of the metabolites decreased in senescent cells as compared to control (CON-1A, 1B and 1C). Increased metabolites in SCC generally came from the TCA and glycolytic cycle while decreased metabolites included a majority of the amino acids measured. Individual metabolite levels associated with various pathway groups and their significance are listed in Table S2.

### 2.7. Adenylate, Guanylate Energy Charge and Creatine Levels in Senescent HCT-116 Cells (SCC) 

**Figure 4:**
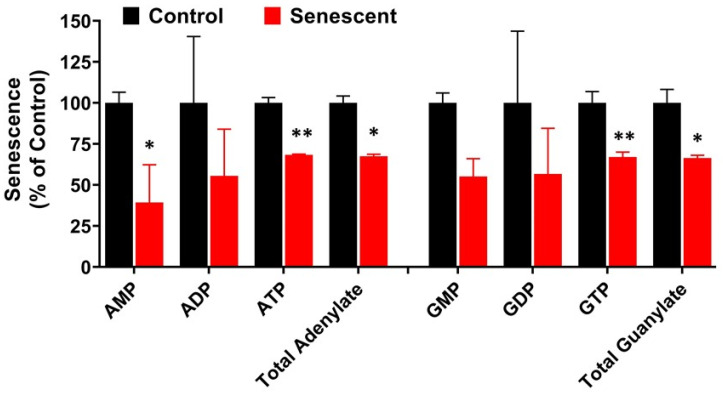
Levels of energy charge adenylate and guanylate in control and senescent HCT-116 cells. Metabolites in senescent cells are expressed as percent of control based on protein concentration of samples. Results are ± SD of triplicate samples. * *p* value < 0.05; ** *p* value < 0.01. Actual values obtained from analysis (nmoles/g protein) including *p* values and significance is presented in Table S2.

Adenylate and guanylate energy charge levels are generally considered as biochemical indicators of cellular metabolic activity, and a value of one is an indication of viability [40]. Adenylate energy charge is measured by the ratio of molar concentrations of AMP, ADP and ATP within the cellular compartments. Guanylate energy charge is measured by the ratio of GMP, GDP and GTP within the cellular compartment. In SCC, adenylate and guanylate energy charges were maintained at one despite significantly lower levels of ATP and GTP as well as total adenylates and guanylates (Figure 4; Table S2). Another indicator of cellular energy is phosphocreatine that is linked to adenylates through enzyme creatine phosphokinase. Phosphocreatine levels were lower in SCC (~20% reduced). However, free creatine and total creatine levels did not show any change in SCC in comparison to control cells. Collectively, these results suggest that SCC retain viability and are metabolically active by maintaining sufficient levels of energy sources and by balancing metabolic pathways of aerobic glycolysis and oxidative phosphorylation.

### 2.8. Redox System Metabolites, NAD, NADP and Glutathione in Senescent HCT-116 Cells (SCC) 

**Figure 5:**
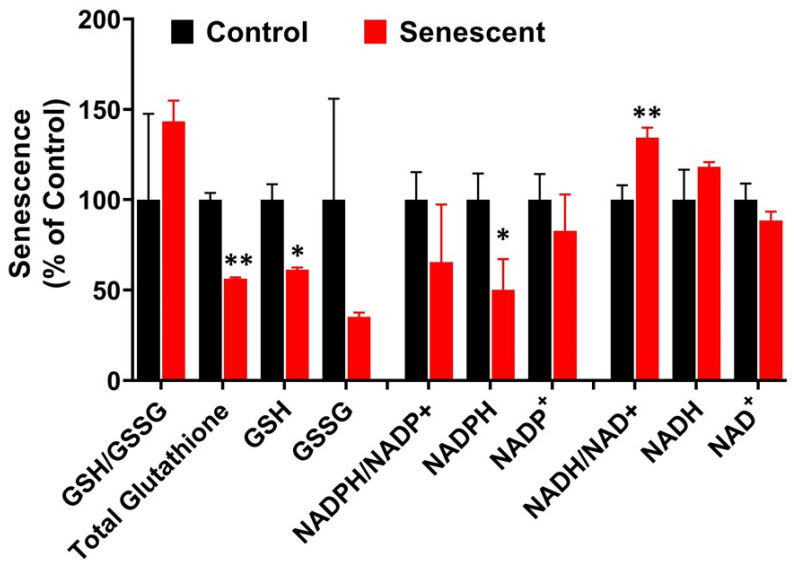
Levels of NAD, NADP and Glutathione metabolites in control and senescent HCT-116 cells. Metabolites in senescent cells are expressed as percent of control based on protein concentration of samples. Results are ± SD of triplicate samples. * *p* value < 0.05; ** *p* value < 0.01. Actual values obtained from analysis (nmoles/g protein) including *p* values and significance is presented in Table S2.

NAD and NADP are critical cofactors in maintaining redox potential associated with many metabolic pathway enzymes, specifically in glycolysis, TCA cycle, pentose phosphate pathway (PPP) and oxidative phosphorylation involved in ATP generation [41]. Levels of NAD, NADH, and NADP were well controlled while NADPH levels were significantly lower in senescent cells (Figure 5; Table S2). In control and SCC, NAD levels were 22,041 and 19,518 while NADP levels were 984 and 814 n moles/g protein respectively (Table S2). In contrast, NADPH levels were significantly reduced in SCC compared to control cells. Total glutathione (GSH + GSSG) and GSH were significantly lower in SCC compared to control cells (Figure 5; Table S2). 

### 2.9. Aerobic Glycolysis with Accumulation of Pyruvate and Lactate in Senescent HCT-116 Cells (SCC) 

**Figure 6:**
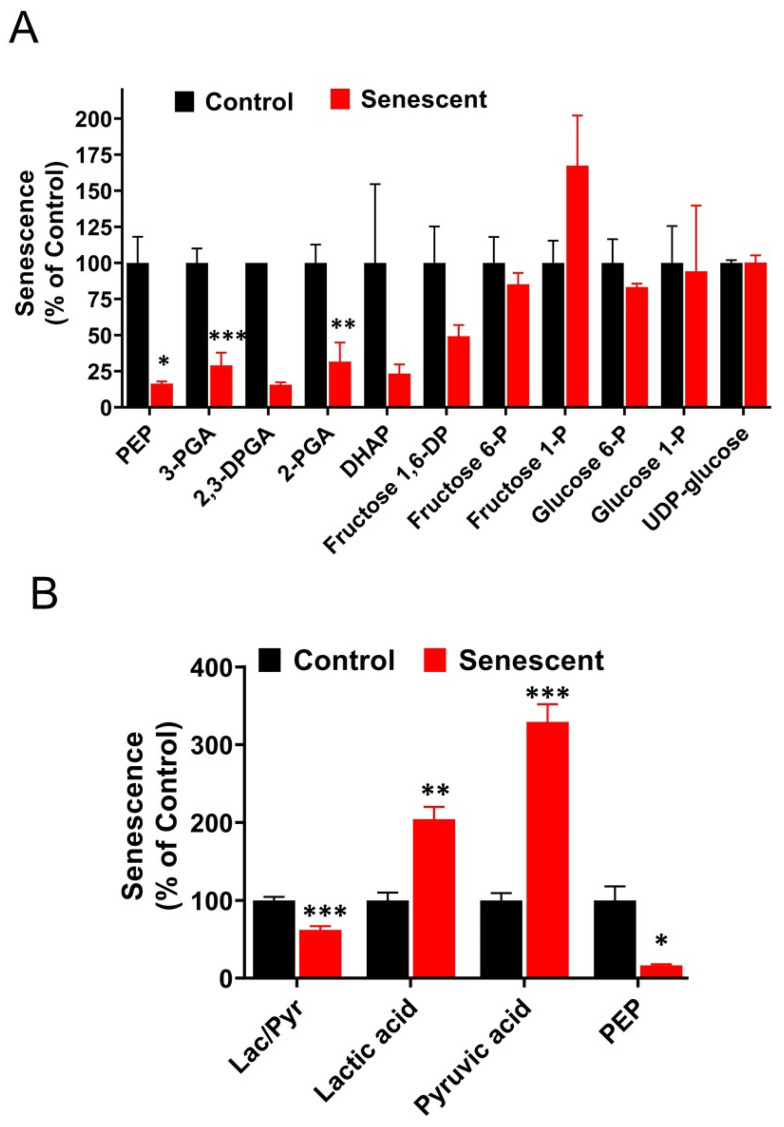
(**A**). Levels of glycolysis-related metabolites in control and senescent HCT-116 cells. Metabolites in senescent cells are expressed as percent of control based on protein concentration of samples. Results are ± SD of triplicate samples. (**B**). Levels of pyruvate and lactate and their ratio in control and senescent HCT-116 cells. * *p* value < 0.05; ** *p* value < 0.01, *** *p* value < 0.001. Actual values obtained from analysis (nmoles/g protein) including *p* values and significance are presented in Table S2.

It is important to note here that culture media contains 4500 mg/L of glucose, 4 mM glutamine and 1 mM sodium pyruvate. Levels of glycolysis metabolites including pyruvate and lactate in control and SCC are presented in Figure 6. Results were expressed as percent of control and actual values are given in Table S2. UDP-glucose, glucose1-phosphate, glucose 6-phosphate and fructose 6-phosphate were unchanged indicating initial steps in glucose entry into glycolysis were not affected in SCC (Figure 6A; Table S2). Many intermediates in the following steps starting from fructose 1,6-diphosphate to phosphoglyceric acid and phosphoenolpyruvic acid were significantly or substantially lower in SCC cells.

This decrease could be due to excessive use of these intermediates for pyruvate and lactate production and/or glycolytic regulatory actions of phosphofructokinase and aldolase enzymes. Levels of phosphoenolpyruvic acid (PEP), pyruvic acid and lactic acid in SCC are presented as percent of control (Figure 6B; Table S2). While PEP levels decreased significantly (*p* < 0.05), both pyruvic acid and lactic acid increased significantly in senescent cells (*p* < 0.001). Pyruvic acid and lactic acid levels in SCC were 12,685 and 642,905 n moles/g protein, respectively. Intracellular pyruvate and lactate increased by 3- and 2-fold, respectively, in SCC compared to control cells.

### 2.10. TCA (Citric Acid) Cycle Metabolites in Senescent HCT-116 Cells

**Figure 7:**
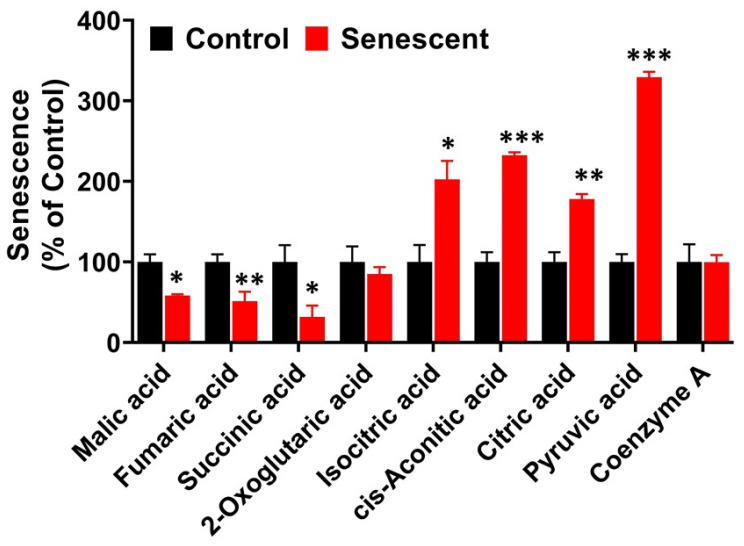
Levels of TCA (citric acid) cycle-related metabolites in control and senescent HCT-116 cells. Metabolites in senescent cells are expressed as percent of control based on protein concentration of samples. Results are ± SD of triplicate samples. Actual values obtained from analysis (nmoles/g protein) including *p* values and significance are presented in Table S2. * *p* value < 0.05; ** *p* value < 0.01, *** *p* value < 0.001.

The entry of cytosolic pyruvate into mitochondrial compartment and conversion to citric acid and isocitric acid through isocitrate dehydrogenase proceeded completely unabated in the presence of adequate coenzyme A levels, as indicated by the levels of these metabolites, (Figure 7, Table S2). Acetyl CoA and acetoacetyl CoA was not detected both in control and SCC, suggesting that these cofactors were efficiently utilized (Table S2). Further steps in the TCA cycle from levels of succinic acid to malic acid decreased significantly in SCC (Figure 7, Table S2). The absolute levels of citric acid and malic acid, in both proliferating and senescent cells, were significantly higher than other TCA cycle intermediates (Table S2). 

### 2.11. Pentose Phosphate Pathway Metabolites in Senescent HCT-116 Cells (SCC) 

**Figure 8:**
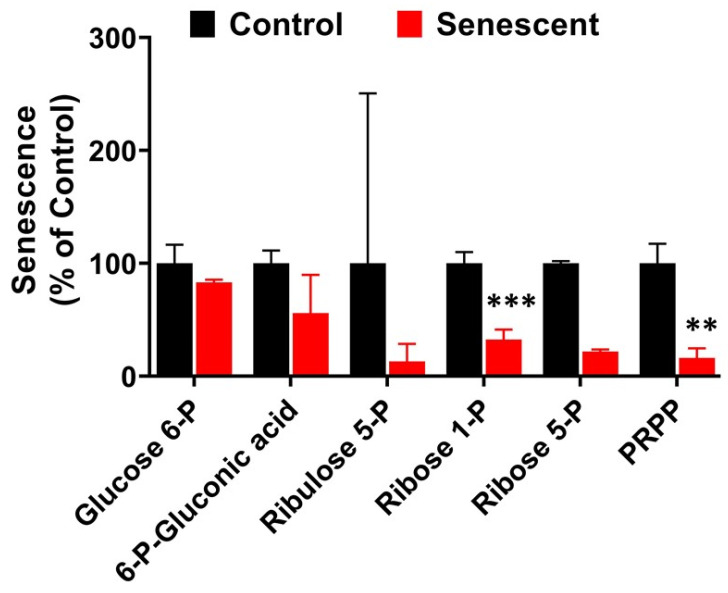
Levels of pentose phosphate pathway-related metabolites in control and senescent HCT-116 cells. Metabolites in senescent cells are expressed as percent of control based on protein concentration of samples. Some metabolites were undetectable in both control and senescent cells. Results are ± SD of triplicate samples. Actual values obtained from analysis (nmoles/g protein) including *p* values and significance is presented in Table S2. ** *p* value < 0.01, *** *p* value < 0.001.

Next, we examined the levels of PPP intermediates in SCC in comparison to control HCT-116 cells (Figure 8 and Table S2). PPP metabolites starting from 6-phosphogluconic acid were substantially lower in senescent cells. Levels of ribose 1 and PRPP (5-Phospho-D ribose 1-pyrophosphoric acid) were significantly lower in SCC in comparison to control HCT-116 cells (70% and 80%, respectively). Other intermediates, xylulose 5-phosphate, sedoheptulose 7-phosphate and erythrose 4-phosphate were not detectable in both control and SCC. 

### 2.12. Levels of Amino Acids Decreased Significantly in Senescent HCT-116 Cells (SCC) 

**Figure 9:**
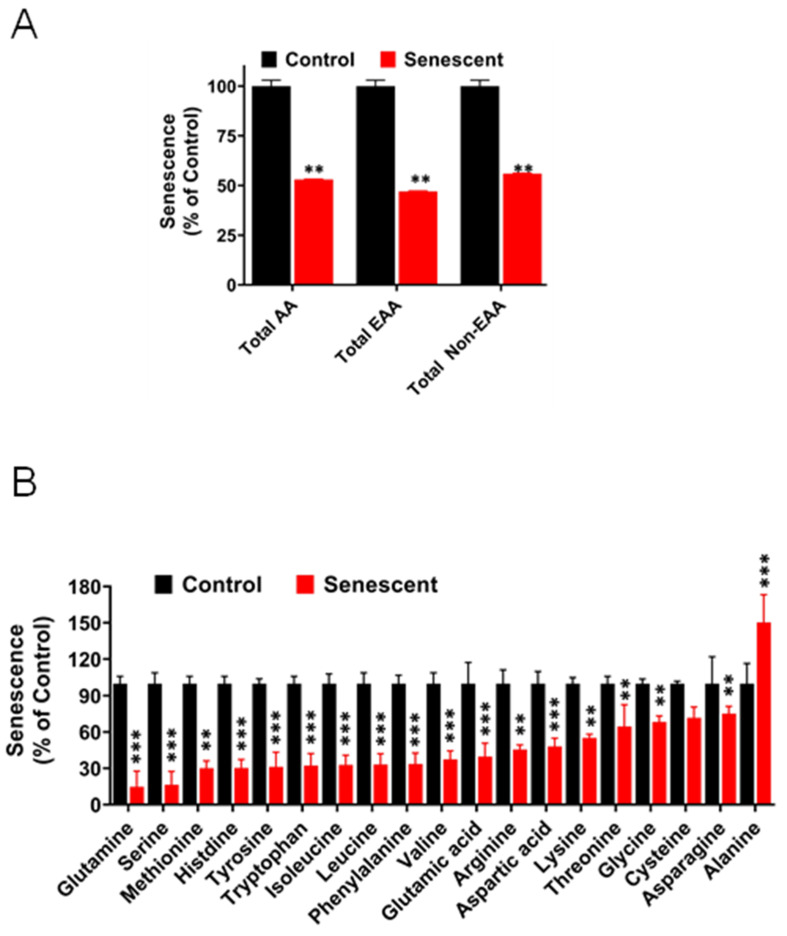
(**A**,**B**). Levels of amino acids in control and senescent HCT-116 cells. (**A**) Total amino acids (AA), essential amino acids (EAA), and nonessential amino acids (non-EEA) in control and senescent cells. (**B**) individual amino acids levels in control and senescent cells. Metabolites in senescent cells are expressed as percent of control based on protein concentration of samples. Results are ± SD of triplicate samples. Actual values obtained from analysis (nmoles/g protein) including *p* values and significance is presented in Table S2. ** *p* value < 0.01, *** *p* value < 0.001.

Metabolomic analysis of amino acids in control and SCC cells are presented as groups based on functional or structural criteria (Figure 9A) and as individual amino acids (Figure 9B and Table S2). Most amino acids, classified as functional groups, decreased significantly (*p* < 0.01–0.001) in senescent cells in comparison to control cells (Figure 9A). Two amino acids increased, alanine and proline. Alanine increased significantly. Proline was not detected in control cells but was found in high quantities in SCC (Table S2). Glutamine levels in SCC were about seven-fold less than in control cells, even though excess glutamine was present in the cell culture medium, suggesting that glutamine consumption was very high in SCC. In terms of absolute values expressed as nmoles/g protein, levels of glutamic acid and glutamine were highest followed by glycine and threonine (Table S2).

### 2.13. Polyamine Metabolites Are Differentially Regulated in Senescent HCT-116 Cells (SCC)

**Figure 10:**
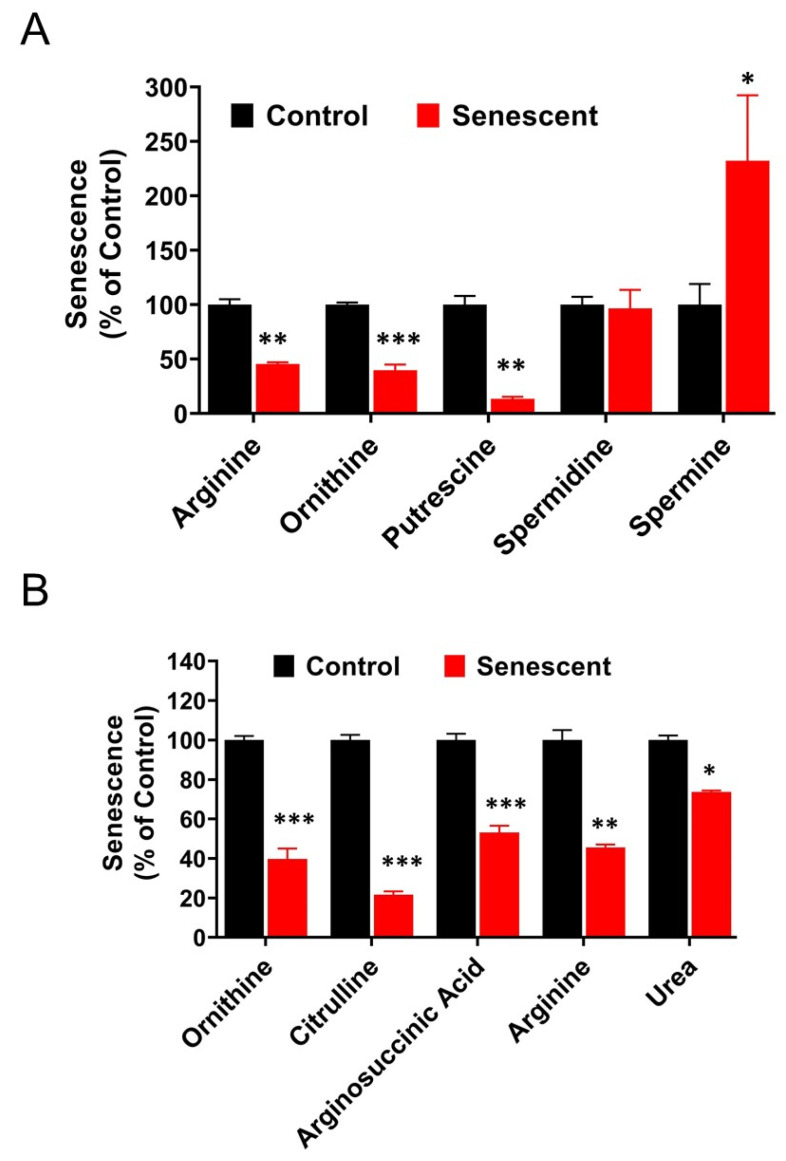
Levels of Polyamine and Urea metabolites in control and senescent HCT-116 cells. (**A**) Polyamine metabolites in control and senescent HCT-116 cells. (**B**) Urea cycle metabolites in control and senescent HCT116 cells. Metabolites in senescent cells are expressed as percent of control based on protein concentration of samples. Actual values obtained from analysis (nmoles/g protein) including *p* values and significance is presented in Table S2. * *p* < 0.05, ** *p* < 0.01, *** *p* < 0.001.

We observed changes in several metabolites involved in the regulation of polyamine levels in senescent cells (Figure 10A). Arginine, putrescine, and ornithine levels were significantly (*p* < 0.01–0.001) decreased in senescent cells compared to control cells (55%, 60%, and 87%, respectively). Whereas spermidine, an intermediate metabolite between putrescene and spermine, was unchanged. In contrast, spermine, was significantly elevated (232%, * *p* < 0.05). In addition, metabolites involved in the urea cycle were also significantly decreased (Figure 10B).

Figure S6 shows a complete pathway analysis of the metabolites studied in this report. Included in the figure are metabolites which were not measured in this report but which make up part of the various pathway listed. Of the metabolites shown in Table S2, 9 were increased, 37 were decreased, and 29 showed no change. 

## 3. Discussion

Cellular senescence is considered as a tumor suppressor program and is reported to be predictive of treatment outcomes in many cancers [1,2,3,6,7,9,11,38,42,43,44]. In response to activated oncogenes and DNA damage due to chemotherapy and radiotherapy, some of the cells may acquire state of senescence when cell cycle is permanently arrested. Colorectal cancer patients achieved significantly longer progressive-free survival when presenting with more senescence positive cells in tumors before therapy [1,24]. However, this notion is now changing, and cellular senescence is viewed as a double-edged sword in cancer progression [43,45] so that the therapeutic outcome is improved, but might, on the other hand, also be induced unintentionally in surrounding normal cells, causing inflammation, secondary tumors, and cancer relapse. The present in vitro study with human colon cancer HCT-116 cells aimed to determine distinct reprograming of secretory (SASP) and metabolic (Metabolomic) pathways upon acquiring IR-induced senescence and how this would affect radiotherapy treatment. In our current report, senescence was induced in proliferating HCT-116 cells by 20 Gy IR and both metabolic components and secretory proteins were analyzed. 

Among regulators of inflammation, only two proteins, secretion of IL-1α and IL-27, were significantly increased in senescent cells. IL-1α is a pro-inflammatory cytokine targeting both lymphoid and non-lymphoid cells. IL-1α signals through NF-kB and activates expression of many genes to initiate a cascade of inflammatory processes including TH17 lymphocyte differentiation [46]. In contrast, IL-27 acts as anti-inflammatory molecule by inhibiting TH17 lymphocyte maturation and promoting TH1 cell differentiation [46]. The elevated levels of chemokines play a critical role in the recruitment of inflammatory cells such as macrophages, neutrophils and lymphocytes into tumor microenvironment, which in turn secrete many molecules that influence cancer cell pathophysiology [47]. The interplay of these molecules regulates overall tumor immune regulation and surveillance influencing tumor microenvironment and cancer progression or suppression. 

In our study, in contrast to growth factors, we observed a significantly decreased secretion of VEGF-A by SSC compared to control cells (Figure 2E). A balancing act of angiogenic factors VEGF-A, endothelin, PLGF, HB-EGF, and others would dictate the final outcome of neovascularization within the tumors [48]. Since VEGF-A is already secreted at high levels by control HCT116 cells (Table S1), lower levels of VEGF-A could potentially negatively regulate angiogenesis within these tumors. The changes observed in angiogenic molecules in SCC cells will require further investigation to understand their exact functional implications in tumor progression. 

In the current study we also analyzed the effect of senescent cells on the expression of key ECM proteins. Matrix metalloproteases (MMPs) and tissue inhibitors of MMPs (TIMPs) play important roles in regulating extracellular matrix (ECM) turnover and maintaining structural integrity of ECM [48,49]. Dissolution of ECM results in loosening of tumor tissue and release of single and clusters of cancer cells from tumors into circulation and migration to secondary tissue sites for metastasis. Augmented secretion of TIMP-1, 2 and 3 and a relatively small increase in MMP-1 by SSC compared to control cells (Figure 2D) would suggest a reduction in ECM degradation in the presence of increased SSC. In summary, the secretory phenotype of senescent cells four days post-IR suggests key distinct changes in the inflammatory molecules, chemokines and ECM proteins. Further analysis of senescent cells past 4 days could depict the dynamic changes in the SASP to assess the terminal SASP phenotype of the senescent tumor cells and its implication in the tumor microenvironment and outcome to radiotherapy. 

Metabolic reprogramming is a salient feature of proliferating tumor cells to meet the altered metabolic requirements needed to adapt to a continuously evolving tumor microenvironment [32,33,34,50,51]. Aerobic glycolysis is the hallmark of tumor cells, and this phenomenon is widely known as the Warburg effect [50,51,52]. IR treatment imposes DNA damage resulting in a variety of cell death mechanisms [19,20,24,25,26,35]. However, some of the cells may become senescent where the cell cycle is arrested but cells retain viability with altered morphology, physiological processes and metabolic patterns [14,20,21,27]. A number of recent studies report significant changes in cellular metabolism during OIS (oncogene-induced senescence), including alterations in nucleotide, glucose, and mitochondrial metabolism and autophagy [53]. Furthermore, studies have shown that pharmacological targeting of metabolic demands using 2DG and bafilomycin on TIS (therapy-induced senescence) induction in vivo leads to tumor regression and improved treatment outcomes [54]. Therefore, information regarding alterations in metabolic pathways in senescent cancer cells due to different external stimuli may have importance in the design of more targeted approaches for killing tumor cells. In this regard, information regarding specific metabolic changes in senescence cells upon IR-induced DNA damage is very limited. The results of our metabolome analysis collectively demonstrate the rewiring of the metabolism in SSC HCT-116 cells with enhanced aerobic glycolysis, reduced PPP, TCA cycle and amino acid metabolism. 

Our studies indicated that senescent HCT-116 cells were in metabolically stable and in active state maintaining normal adenylate and guanylate energy charge levels, despite lower levels of ATP and GTP (Figure 4) [40]. This is further supported by creatine and phosphocreatine levels which are also associated with ATP through creatine kinase activity [33,34]. Levels of nicotine adenine nucleotides, NAD+ and NADP+ remained unchanged in senescent HCT-116 cells. NADH and NADH/NAD+ levels in senescent cells were maintained at a healthy level due to enhanced aerobic glycolysis and initial TCA cycle reactions leading to alpha ketoglutarate and succinyl Co A [16,32,33,34,51,52]. In contrast, NADPH levels were significantly decreased due to decreased levels of PPP. Another major molecule associated with oxidation-reduction reactions is GSH [19,32,33,34,41]. In senescent HCT-116 cells, both total GSH (GSH + GSSG) and reduced GSH decreased significantly, indicating that senescent cells could be under oxidative stress. Overall, the reduced levels of ATP, GTP, NADPH and GSH levels suggest that senescent cells after 4 days post-IR have a compromised energy metabolism but remain viable.

The intracellular levels of glycolytic and TCA cycle intermediates in senescent cells depend on cancer cell types, normal epithelial and fibroblasts, and senescence induction mechanisms such as proliferative exhaustion, oncogenes, chemotherapeutic agents or IR. In SSC HCT-116 cells, glycolytic intermediates fructose 1, 6 diphosphate to phosphoenolpyruvate were decreased significantly suggesting potential rate limiting roles of phosphofructokinase and aldolase enzymes [52,55,56]. Molar levels of both pyruvate and lactate were much higher in senescent cells, indicating enhanced aerobic glycolysis in senescent cells compared to proliferating cells. Intracellular lactate is transported to extracellular space by carrier known as MCT-4 while MCT-1 transports extracellular lactate into the cells [34,57,58]. Both the transporters regulate the levels of intracellular lactate to meet the cellular needs and are regulated by the expression of carrier proteins in proliferating and senescent cancer cells. Lactate is considered as a key player in aerobic glycolysis and tumor metabolism by balancing glycolysis and the TCA cycle [33,34,57,58]. Lactate levels increase when glycolytic breakdown of glucose is not followed by efficient mitochondrial oxidation. Tumor lactate is thought to be an indicator for predicting metastasis by influencing the migration of cells and cell clusters from tumors [58]. In contrast, the current study shows an increase in lactate but a decrease in VEGF and no significant effect on ECM degradation in SCC, indicating that SASP tumor cells are undergoing changes that could ultimately determine the fate of non-cancerous tumor cells in vivo.

Pyruvate plays critical roles in cancer cell metabolism by being at the crossroads of glycolysis, citric acid cycle and amino acid metabolic pathways [32,33,34,55]. Metabolome analysis in senescent cells showed increased levels of citric acid suggesting transport of pyruvate into mitochondria and availability of acetyl CoA for the synthesis of citrate were not the limiting factors. While citric acid levels were elevated in senescent HCT-116 cells, other intermediates in subsequent steps, succinate, fumarate and malate decreased significantly. These could be due to rate limiting actions of succinate dehydrogenase, fumarase and malate dehydrogenase enzymes during IR-induced senescence. Alteration in these metabolites could also be due to the utilization of 2-ketoglutarate and oxaloacetate for the synthesis of several amino acids to meet the demands of enhanced protein synthesis during senescence [33,34]. In contrast, all citric acid cycle intermediates decreased in proliferation-exhausted senescent normal NHOF-1 oral fibroblasts [36,37]. In our study, enhanced aerobic glycolysis is in agreement with metabolic effects seen in chemotherapy-induced senescent tumor cells [54]. After senescence-inducing chemotherapy, therapy-induced senescence (TIS) -competent lymphomas, but not TIS-incompetent Suv39h1– lymphomas, show increased glucose utilization and much higher ATP production [54]. However, in HCT-116 cells, we observed decreased levels of ATP (Figure 4) and did not appear to be under energy stress. These data suggest that metabolic patterns depend on multiple factors such as cell types, cell culture conditions and mechanisms of senescence induction. 

The pentose phosphate pathway plays critical roles in generating NADPH for many metabolic, oxidation-reduction and nucleotide synthesis reactions [32,33,34,41]. In senescent HCT-116 cells, PPP intermediates, ribose and ribulose phosphates, and phosphoribosyl pyrophosphate (PRPP) decreased significantly. Consistent with reduced PPP activity, NADPH levels were maintained at significantly lower levels. PRPP has critical roles in purine and pyrimidine nucleotide, NAD, histidine and tryptophan biosynthesis [33,34,41]. This indicates that the DNA damage after IR exposure in senescent cells may not be repaired due to significant reduction in the nucleotide biosynthesis. In contrast to our results, in proliferation exhaustion senescence in NHOF-1 cells, all PPP intermediates increased significantly [36]. These results indicate that distinct metabolic patterns of PPP in senescent cells may partly depend on the nature of external or internal stimuli. 

In addition to SASP alterations, metabolome analysis of amino acids in senescent cells indicated a very clear picture of the excessive use of amino acids for protein metabolism. In addition to extracellular amino acids available from the growth medium, many amino acids are synthesized intracellularly from metabolic intermediates [32,33,34]. Alanine and proline were the only amino acids that were significantly increased in senescent HCT116 cells, while the levels of all other amino acid decreased very significantly (Table S2, Figure 9B). It is conceivable that high levels of alanine in senescent cells could be due to synthesis from pyruvate accumulated in senescent cells. On the other hand, 2, 3-phosphoglycerate, one of the sources for serine, glycine and cysteine production, was significantly lower, probably due to usage for amino acid synthesis and accelerated glycolysis in senescent cells. 2-ketoglutarate is one of the major sources for intracellular production of glutamate, glutamine, proline and arginine amino acids [32,33,34]. In senescent HCT-116 cells, levels of isocitrate were significantly higher while succinate was significantly lower in comparison to ketoglutarate, an intermediate molecule, suggesting efficient usage of ketoglutarate for amino acid synthesis. Proline was the only amino acid that was detected at high levels in senescent cells while being undetected in control proliferating cells (Table S2). Proline and hydroxyproline play essential roles in the synthesis of extracellular matrix (ECM) components containing collagen molecules [32,33,34,48,49]. To further validate the high demand for amino acids for synthesizing the proteins in the cells by the SCC cells, we used a protein synthesis inhibitor cycloheximide to inhibit this distinct metabolic phenotype. Interestingly, SCC cells treated with cycloheximide showed a markedly reduced percentage of senescent cells compared to the untreated SCC cells (data not shown). Once the cycloheximide was washed away, the senescent phenotype of the irradiated cells was restored as seen by enhanced beta gal staining (data not shown). We believe this preliminary observation with a general protein synthesis blocker requires further studies. 

Compared to proliferating HCT-116 cells, levels of citrulline, ornithine and putrescene were significantly lower in senescent cells, while spermine and spermidine levels were not affected. Polyamines are considered critical for tumor cell growth [32,33,34,59] and their low levels in senescent HCT-116 cells suggest their possible role in the inhibition of cellular proliferation. In addition to metabolic alterations, intracellular-extracellular traffic of these molecules may contribute to the intracellular levels of putrescene, spermine, spermidine and their by-products. 

The changes observed in both metabolic pathways and secretory pathways in IR-induced SSC are indeed complex. Further, the mechanisms by which malignant cells undergo IR-induced senescence and how they may influence tumor response or regrowth post-IR treatment is both complex and a challenge to evaluate. With radiotherapy now transitioning, when possible, to stereotactic body radiation therapy (SBRT), precisely delivering large doses of IR to the tumor, senescence may well contribute to the overall treatment response. IR-induced SASP from senescent cells may have the potential to influence cancer suppression or progression tumors [8,60]. Previous studies have shown that PARP inhibitor veliparib radiosensitizes tumor models through the induction of senescence characterized by a modified immunostimulatory SASP and activation of an antitumor adaptive immune response [61]. This work suggests a route to enhancing the benefits of radiotherapy, whereby senescent tumor cells act as a cancer vaccine to target the tumor [61]. In conclusion, although some evidence suggests a connection between senescence and radiotherapy outcome [62,63,64], experiments are further needed to better characterize the molecular and functional links between these two programs, especially in in vivo models in which data are currently very few. 

Our in vitro senescence model could be useful as a platform to further evaluate the effectiveness of novel molecules such as senolytics, protein synthesis inhibitors, inhibitors of specific amino acid transporters, glycolytic inhibitors and nutraceuticals on senescence processes by SASP and transcriptome analysis. Future metabolism studies coupled with other investigations regarding the role of senescence in chemo-radiation [65] may provide a better understanding of how senescence influences tumor response following IR treatment in the clinical setting. 

## 4. Material & Methods

### 4.1. Cell Cultures 

HCT-116 (ATCC CCL-247), SCCVII (mouse squamous carcinoma cells) were used. Most of the studies were conducted using HCT-116, a human colon colorectal carcinoma cell line derived from an adult male Caucasian colon [66]. This cell line has a mutation in codon 13 of the KRAS (G13D) proto-oncogene and PIK3CA (H147R). This cell line expresses wild type genes for TP53, PTEN and BRAF [67]. HCT-116 cells are positive for carcinoembryonic antigen (CEA) and keratin. HCT-116 cells were grown in Dulbecco’s Modified Eagle’s medium with high glucose (HyClone # SH30022-01), containing 4500 mg/L glucose and 4 mM L-glutamine was supplemented with heat-inactivated 10% fetal bovine serum, sodium pyruvate 1 mM, penicillin (100 units/mL) and streptomycin (100 μg/mL). SCCVII cells (derived from mouse oral squamous cell carcinoma) were grown in MEM medium supplemented with heat-inactivated 10% fetal calf serum, penicillin and streptomycin. All cell cultures were grown and maintained at 37 °C in an atmosphere of 5% CO_2_ and 95% air. 

### 4.2. Radiation Treatments

HCT-116 and other cells were plated in 60 mm (30–50 K cells) or 100 mm (100 K cells) dishes depending on experiments. After three days, the medium was replaced with a fresh medium and exposed to ionizing radiation (IR) of 2000 cGy (20 Gy) using X-RAD 320 (Precision X-Ray Inc, N. Branford, CT, USA). Any changes in radiation dosage are indicated in the legends to the figures. After IR treatment, media were not changed until the conclusion of the experiment. Any deviation from these conditions is indicated in detail in the legends to figures. 

### 4.3. Senescence-Associated Beta Galactosidase (SA-β Gal) Detection

Commercially available SA-β gal staining kits were used for detecting senescence in cells (Biovision (# K320-2500) Milpitas, CA-95935, USA or Abcam (# ab 65351). HCT-116 cells and other cells grown in 35, 60 or 100 mm culture dishes or glass slides were subjected to IR. Cells were typically used after 4 or 5 days post-IR for detecting SA-β gal according to the procedure recommended by the manufacturer. In brief, culture media were removed and washed once with PBS and incubated with fixative solution for 3–5 min, followed by washing twice with PBS. SA β-gal staining solution containing X-GAL (1 mg/mL) dissolved in DMF (dimethyl formamide) was added to fixed cells and incubated at 37 °C in a non-CO_2_ incubator. A blue color was observed after 24 h and the intensity of the color was enhanced after further incubation. Blue stained and unstained cells were counted, and photographs were taken using a KEYENCE microscope (BIOREVO, BZ-9000, Osaka, Japan). Cell counting was performed typically in 10 or more visual fields at different locations in the same dish and mean and the percentage of SA-β gal positive cells were calculated. 

### 4.4. Protein Extraction and Quantitation

Cultures were washed with ice-cold PBS twice and cells were collected into ice-cold PBS using a cell scraper. The cell suspension was pelleted by centrifugation at 2000 rpm for 10 min at 4 °C. The cell pellets were frozen at −70 °C until processed. Cell pellets were thawed on ice and incubated with RIPA buffer containing protease and phosphatase inhibitors for 30 min. The sample tubes were centrifuged for 30 min at 15 k rpm and the supernatants collected were used for protein immunoblot analysis. Quantitation of proteins was done by using Bio-Rad DC protein assay or by Pierce BCA protein assay reagents.

### 4.5. Western Immunoblot Analysis of Proteins

Denatured protein samples were separated on SDS PAGE 4–20% NOVEX gels and transferred to nitrocellulose membranes using iBlot dry blotting system (Invitrogen, Carlsbad, CA, USA). After blocking in 2% solution of fat free dry milk powder, the membranes were incubated in the relevant primary antibodies followed by washing, and further incubation with HRP conjugated secondary antibodies. The membranes were then washed and developed by using chemiluminescence reagents (Thermo Scientific, Waltham, MA, USA). Protein band images were captured with Fluor Chem HD2 imager (Alpha Innotech, San Leandro, CA, USA) and processed using image analyzer software. Blots were stripped and re-probed with actin or HSC 70 to confirm that an equal amount of protein was loaded into each well. The following primary antibodies were used for western blots: p53 (Abcam #ab 32049), p21 (BD Pharmingen), Actin (Millipore # MAB 1501R), HSC 70 (Santa Cruz #J2212). 

### 4.6. Radiation Treatments and Collection of Culture Supernatant Samples

HCT-116 cells were plated in 60 mm (40–50 K cells) dishes (5 mL/dish). After three days in culture, the medium was replaced with fresh medium and exposed to IR of 2000 cGy (20 Gy). After 24 h culture supernatants from control (non-IR) were collected, clarified by centrifugation at 2000 rpm for 10 min and frozen and stored at −70 °C. After 72 h, media from IR exposed cultures were removed and replaced with fresh medium. Culture supernatants from these dishes were collected after 24 h and processed as above and frozen at −70 °C. Corresponding blanks were made by keeping fresh culture medium in culture dishes under similar conditions but without cells. The same batch of cell cultures treated under similar conditions were used for cell counts, SA-β gal staining and protein estimation.

### 4.7. Multiplex Luminescence Analysis of Secreted Proteins in Culture Supernatants 

Multiplex luminescence assays for several secreted protein analytes were performed by EVE technologies Inc. (Alberta, Canada) using their standard propriety protocols. The following panel groups were selected for luminescence assays; Human-Cytokine-48-Plex, Human-Angiogenesis-Growth-Factor-17-Plex, Human-MMP-9-Plex-TIMP-4-Plex and TGF-beta-3-Plex. All four panels include 81 proteins typically secreted by cells in culture. All results are expressed as pg protein analyte per mg of cellular protein. Complete details of all analytes are presented in figures and supplements. When medium background (no cells) values were insignificant compared to either the proliferating or senescence cells medium values, these values were subtracted from both the groups to obtain the ratio between the 2 groups. Medium background values, which were not significantly different from the proliferating cells medium values, were not subtracted from either group in order to obtain more reliable ratio values (senescence/proliferating) (Table S1). The ratios reported in Figure 2 were also treated in the same manner. Means and standard deviations were determined using the propagation of error formula for all ratio values reported.

### 4.8. Preparation of Proliferating (Control) and Senescent Cells for Metabolite Extraction

In preliminary studies, we established ideal experimental conditions such as cell numbers, ionizing radiation (IR) dose and post-IR days for inducing senescence in more than 90% cells. Cells (100,000) were plated in 100 mm dishes. After 3 days, cultures were washed to remove floating cells and fed with fresh medium and one batch of cells were exposed to IR dose intensity of 20 Gy. After 24 h, control (non-IR) cells were used for extraction of metabolites as described below. After 96 h, IR-exposed cells (senescent cells) were used for extraction of metabolites. Representative dishes from the same batch of cultures, control and senescent, were used for cell counting, protein quantitation and SA β-gal as described above. Approximately 95% of irradiated HCT-116 cells were found to be SA β-gal positive suggesting 95% of these cells were senescent. In contrast, only less than 2% of control (proliferating) HCT-116 cells were SA-β gal-positive. 

### 4.9. Analysis of Intracellular Metabolites by CE-TOFMS and CE-QqQMS

Methanol-water extraction of metabolites was performed according to the protocol for adherent cells (E-120998) provided by Human Metabolome Technologies Inc (Boston, MA, USA). Culture media were removed from the dishes and washed twice with 5% mannitol solution. After complete aspiration of mannitol solution, cells were extracted with methanol followed by internal standard solution dissolved in Millipore water. Culture extracts were filtered and dried up using a centrifugal evaporator following HMT protocol. Selected component analysis (C-Scope/CARCINOSCOPE) of metabolome profiles of HCT-116 cells was performed by CE-TOFMS and CE-QqQMS methods and used for analyzing cationic and anionic metabolites (54 cation and 62 anion mode, respectively). Hierarchial cluster analysis (HCA) and principal component analysis (PCA) were performed by statistical analysis software developed by HMT. Detected metabolites were plotted on metabolic pathway maps using the VANTED (Visualization and Analysis of Networks containing Experimental Data) program developed by HMT Inc. Levels of metabolites were expressed as nmoles/gram protein.

## Data Availability

The data presented in this study are available in this article and supplementary material.

## Supplementary Materials

The following are available online at https://www.mdpi.com/article/10.3390/ijms22094835/s1. Figure S1: Radiation induced senescence in tumor cell lines. Figure S2: Photomicrographs of HCT-116 cells 7 days post-IR (20 Gy). Figure S3. Levels of adherent senescent HCT-116 cells following 20 Gy post-IR. Figure S4. Effects of radiation dose (Gy) on the expression of senescent marker proteins. Figure S5. Levels of PRPP synthetase pathway metabolites in control and senescent HCT-116 cells. Figure S6. Schematic presentation of complete pathway analysis of the relative levels of metabolites in IR-induced senescent HCT-116 cells compared to proliferating cells (control) studied in this report. Table S1. Proteins secreted by proliferating and senescent HCT116 cells. Table S2. Metabolite Concentrations in Proliferating and Senescent HCT116 cells.

## Abbreviations

CRC	Colorectal cancer
ECM	Extracellular matrix CRC
HCT-116	Human colon cancer cells
SCC	Senescent cancer cells
SCCVII	Mouse squamous cell carcinoma cells
IR	Ionizing radiation
cGy	cGray units, radiation intensity
SA- β-Gal	Senescence-associated β-galactosidase
NAD	Nicotinamide adenine dinucleotide
NADH	Nicotinamide adenine dinucleotide hydride
TCA	Tricarboxylic acid cycle (citric acid cycle)
PPP	Pentose phosphate pathway
Aldolase	Fructose, 1, 6 biphosphate aldolase
PRPP	5-Phospho-D ribose 1-pyrophosphoric acid
VEGF	Vascular endothelial growth factor
MCT	Monocarboxylate transporter
SASP	Senescence-associated secretory phenotype
TGF	Transforming growth factor
PDGF	Platelet derived growth factor
IL	Interleukin
MMPs	Matrix metalloproteases
TIMPs	Tissue inhibitors of MMPs
