# Supplementary material for: Radiation-Induced Senescence Reprograms Secretory and Metabolic Pathways in Colon Cancer HCT-116 Cells
Source: Int J Mol Sci. 2021 May 3;22(9):4835. doi: 10.3390/ijms22094835 (PMC8124941; doi:10.3390/ijms22094835)
Supplement: Supplementary file 1 [file ijms-22-04835-s001.zip › ijms-1203632-supplementary.pdf]

Supplementary Materials

Radiation induced senescence reprograms secretory and metabolic pathways in colon cancer HCT-116 cells

Chandrasekharam N. Nagineni[1], Sarwat Naz[1], Rajani Choudhuri[1], Gadisetti V.R. Chandramouli[2], Murali C. Krishna[1], Jeffrey R. Brender[1], John A. Cook[1], James B. Mitchell[1]

1. **Radiation Biology Branch, Center for Cancer Research, National Cancer Institute, Bethesda, MD, 20892, USA.**
2. **Genepria Consulting Inc. Columbia, MD, 21046, USA.**

naginenic@mail.nih.gov (CNN); sarwat.naz@nih.gov (SN); rajani.choudhuri@nih.gov (RC); murali@helix.nih.gov (MCK); jeffrey.brender@nih.gov (JRB); john.cook@nih.gov (JAC); jbm@helix.nih.gov (JBM); mouli@genepria.com (GVRC)

*** Correspondence: Chandra N. Nagineni, Ph.D. naginenic@mail.nih.gov**

Supplementary Materials: The following are available online at https://www.mdpi.com/article/10.3390/ijms22094835/s1
Figure S1: Radiation induced senescence in tumor cell lines. **Figure S2:** Photomicrographs of HCT-116 cells 7 days post-IR (20 Gy). **Figure S3:** Levels of adherent senescent HCT-116 cells following 20 Gy post-IR. **Figure S4:** Effects of radiation dose (Gy) on the expression of senescent marker proteins. **Figure S5.** Levels of PRPP synthetase pathway metabolites in control and senescent HCT-116 cells. **Figure S6.** Schematic presentation of complete pathway analysis of the relative levels of metabolites in IR-induced senescent HCT-116 cells compared to proliferating cells (control) studied in this report**. Table S1.** Proteins secreted by proliferating and senescent HCT116 cells. **Table S2.** Metabolite Concentrations in Proliferating and Senescent HCT116 cells

Author Contributions: Conceptualization, CNN., SN.,RC., JAC., JBM.; methodology, CNN., SN., RC., JAC., JBM.; funding acquisition, JBM.; resources, JBM.; software and analysis, CNN., GVRC., JAC., RC., SN., JRB.; writing original draft, CNN.; writing-review and editing, CNN., SN., JAC., RC., JRB., MCK., JBM. All authors have read and agreed to the published version of the manuscript.

Funding: This work was supported by the intramural Research Program, Center for Cancer Research, National Cancer Institute, National Institutes of Health.

Acknowledgements: We thank Dr. William Samuel, NEI, NIH for help in the preparation of some figures.

Conflict of Interests: The authors declare no conflict of interest.

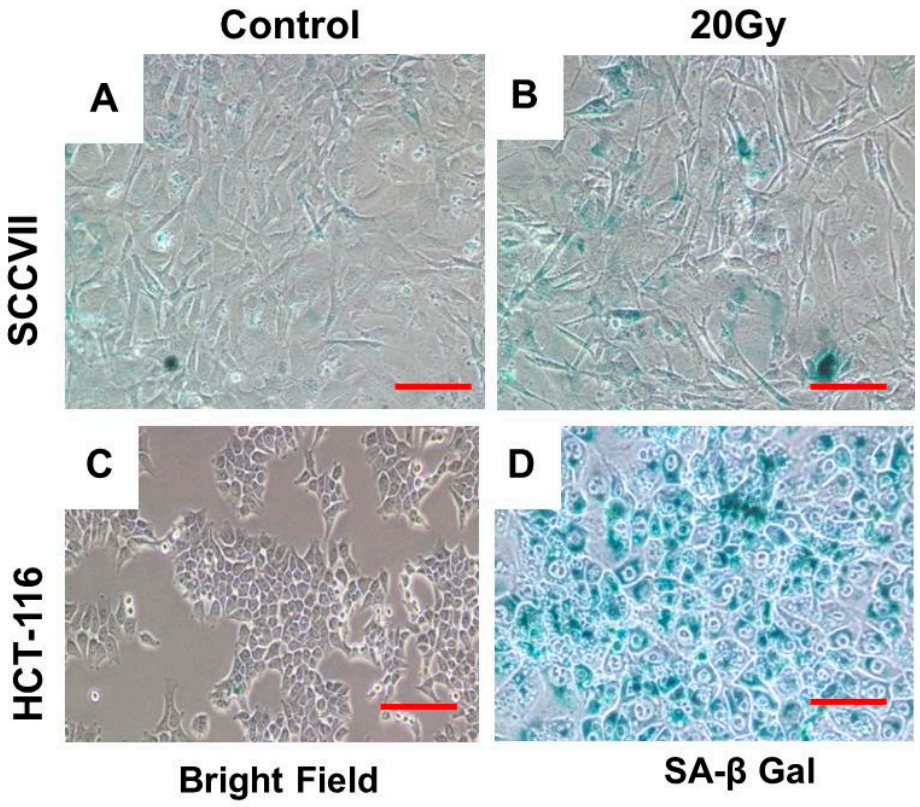

Figure S1: Radiation induced senescence in tumor cell lines. (**A-B**) SCCVII (Murine squamous cell carcinoma) and (**C-D**) HCT116 (human colon cancer) cells were exposed to the indicated dose of IR (Gy). Senescent cells were observed by SA-β gal staining 4 days post IR (**B** and **D**). **A** and **C** represents the phase-contrast microphotographs of control unexposed cells. Scale bar is 100 μm. Magnification is 200X.

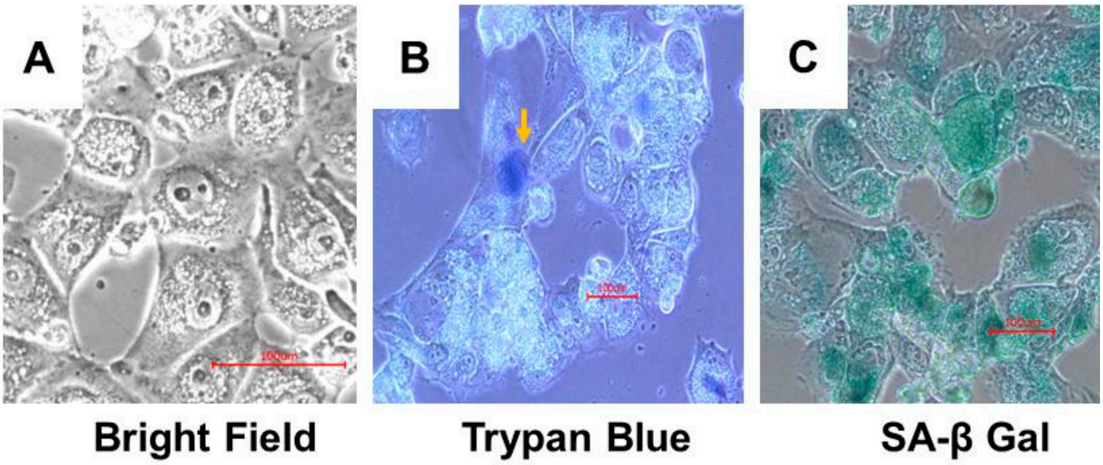

Figure S2: Photomicrographs of HCT-116 cells 7 days post-IR (20 Gy). (**A**) Representative phase-contrast photomicrographs of SCC. (**B**) Trypan blue stained cells. Yellow arrow represents the trypan blue positive dead cells. (**C**) Representative senescent cells observed by SA-β gal staining 7 days post IR (20 Gy). Scale bar is 100 μm. Magnification is 200X.

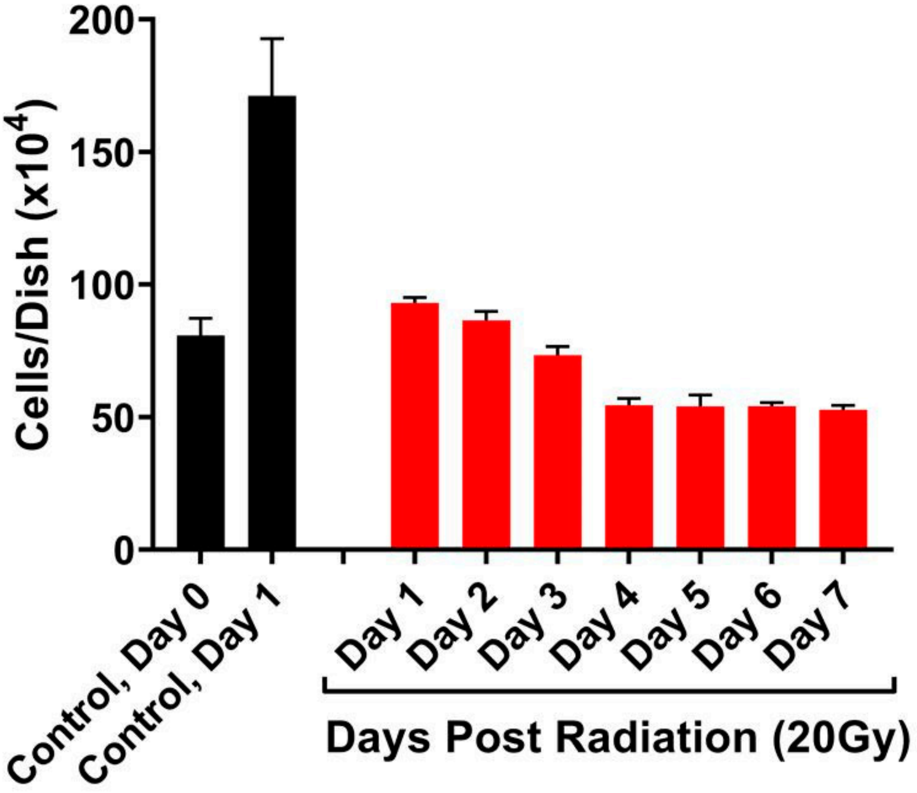

Figure S3. Levels of adherent senescent HCT-116 cells following 20 Gy post-IR. 50k cells were plated in 60 mm culture dishes. After three days media were replaced with fresh medium and exposed to 20 Gy ionizing radiation. On various days (day 0 to 7), cultures were washed with PBS to remove dead and floating cells. Attached cells were dissociated with trypsin-EDTA solution and counted using Beckman Coulter Counter. Day 0 was the day cells were exposed to IR. Triplicate dishes were used for each treatment. Results are from one typical experiment representing two other experiments.

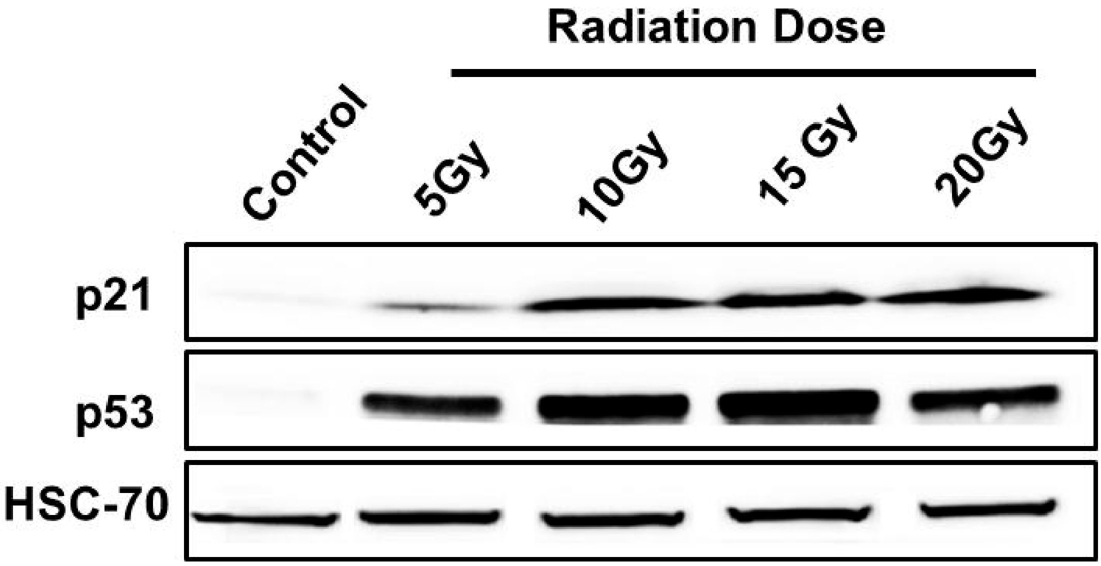

Figure S4. Effects of radiation dose (Gy) on the expression of senescent marker proteins. HCT116 cells were exposed to the indicated dose of radiation. Protein lysates were collected 4 days post radiation. Immunoblot shows the change in the expression of key senescent markers proteins, p53 and p21.

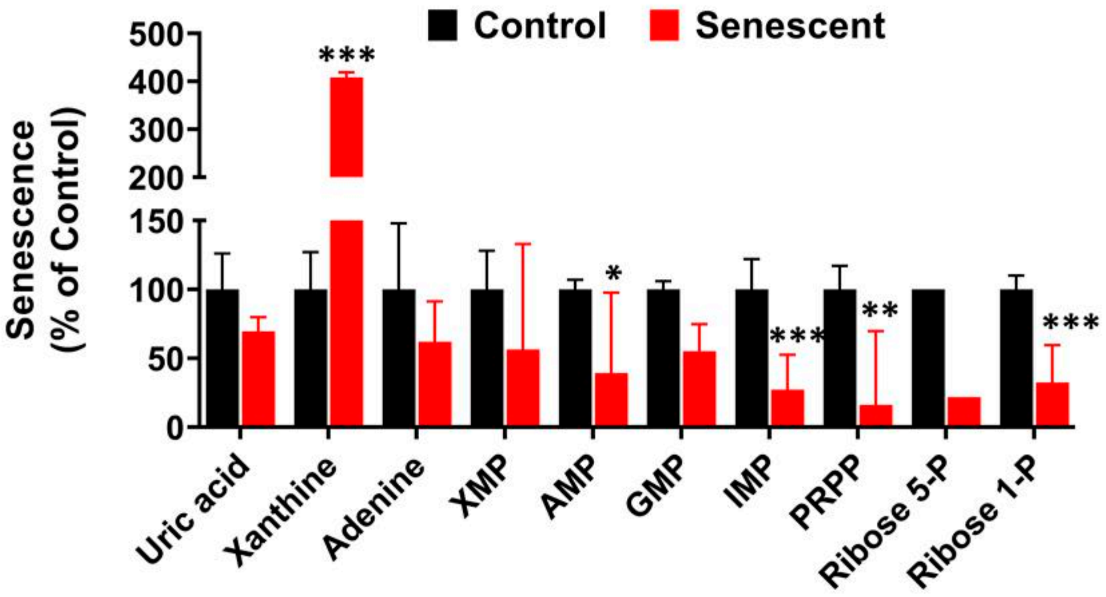

Figure S5. Levels of PRPP synthetase pathway metabolites in control and senescent HCT-116 cells. Differential expression of metabolites in the PRPP synthetase pathway is shown between the control (*black bar*) and senescent cells (*red bar*). The levels of each of the indicated metabolites is normalized to the control and represented as percentage of the control. Each bar represents mean ± standard deviation. The results are representative of samples analyzed in triplicates by the Welch's t-test * $p<0.05$, **<0.001 and ***$p<0.0001$.

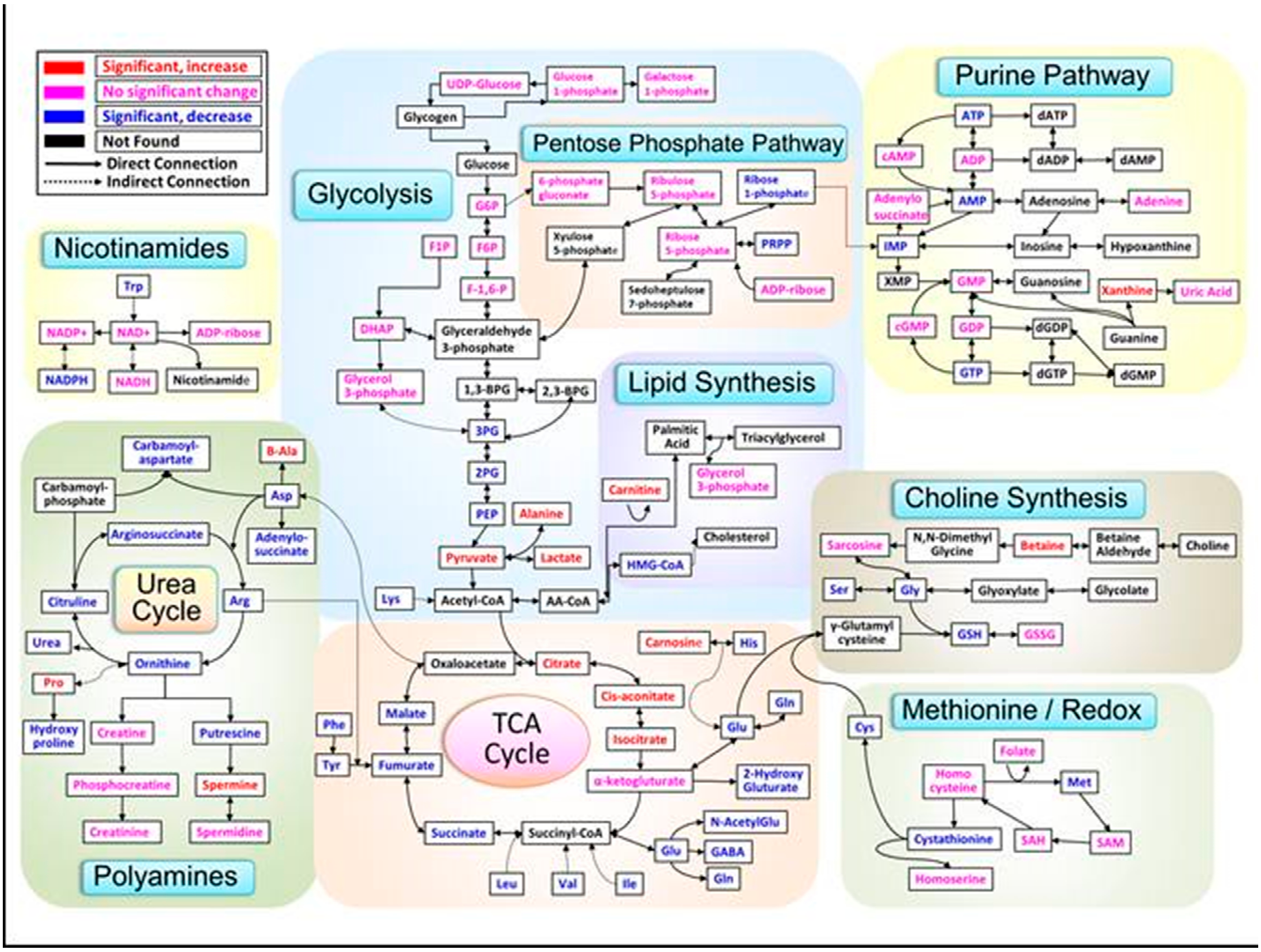

Figure S6. Schematic presentation of complete pathway analysis of the relative levels of metabolites in IR-induced senescent HCT-116 cells compared to proliferating cells (control) studied in this report. Included in the figure are metabolites which were not measured in this report, but which make up part of the various pathways listed. Of the metabolites shown in Table S2, 9 were increased, 37 were decreased, and 29 showed no change. The boxes with **red letters** (Statistically significant increases), **blue letters** (statistically significant decreases) and **pink letters** (no significant changes) indicate metabolite levels in senescent cells in comparison to proliferating cells. White boxes with **black letters** indicate metabolites not detected.

Table S1. Proteins secreted by proliferating and senescent HCT116 cells.

Protein Name	Total Con*** (pg/mg)	Total Sen *** (pg/mg)	Ratio (Sen/Pro)	background control § (pg/mg)
**Angiogenesis Mediators**				
Endothelim-1	15.8(1.8)**	127(6.3)	8	16.7
HB-EGF	31.7(4.3)*	279(8.5)	8.8	2.1
IL-8	216(20)*	3,161(161)	14.6	8.2
PLGF	62(6)*	572(24)	9.3	0.94
VEGF-A	1,766(308)*	675(42)	0.38	2.5
**Interleukins**				
IL-4	4.01(1.1)*	1.55(0.2)	0.39	1.1
IL-6	1.06(0.1)**	2.65(0.2)	2.5	0.94
IL-15	5.09(0.5)**	8.75(1.1)	1.7	4.7
IL-18	1.04(0.1)**	1.48(0.1)	1.4	0.94
IL-27	15.5(13)*	182(13)	11.7	15.9
IL-8 (CXCL8)	216(20)*	3,161(161)	14.6	8.2
**Chemokines**				
CCL-2 (MCP-1)	6.41(0.5)**	13.2(1)	2	5.8
CCL-22 (MDC)	0.92(0.1)**	11.4(1)	12.5	0.94
CXCL-1 (GROα)	18.4(0.9)*	379(20)	20.6	2.1
CXCL-10 (IP-10)	4.1(0.3)**	109(6)	26	4.6
CCL-5 (RANTES)	2.73(0.4)**	500(20)	183	2.1
**MMPs/TIMPs**				
MMP-1	59.1(4.1)**	619(133)	10.5	58.9
TIMP-1	21,098(3,090)*	75,824(6,731)	3.6	1178
TIMP-2	63,730(5,848)*	230,269(14,724)	3.6	1178
TIMP-3	1,419(100)**	36,919(4,801)	26	1178
TIMP-4	55(66)*	65(74)	1.1	24.6
**Growth Factors**				
M-CSF	71.3(4.2)**	319(42)	4.47	61.4
GM-CSF	0.32(0.1)**	46.6(5.5)	144	0.24
TGF-α	153(17)*	980(191)	6.4	2.4
PDGF-αα	778(48)*	2,592(270)	3.3	20
PDGF-ββ/αβ	115(19)*	1,917(219)	16.7	9.1
**Inflammatory mediators**				
IFN-α	15(1.8)**	10.4(0.4)**	0.69	21
IFN-γ	2(0.1)**	1.4(0.1)**	0.71	1.8
TNF-α	11.9(1.5)**	10.2(0.1)	0.86	10.1
TNF-β	36.1(7.7)*	14.6(1.3)	0.4	2.1
IL-1α	7.5(0.3)**	27.4(9)	3.6	7.3
IL-1β	3.6(1.8)**	2.1(0.4)**	0.58	3.1

Concentrations corrected for background medium levels. ** Not different from background medium levels. Background was not subtracted from either control or Senescence cells. *** Values in () are standard deviations. Con -- Control, Sen – Senescence. (pg/mg) – picogram chemokine/mg protein. § Background of medium blank using control cell numbers for calculation. Essentially, medium blank (with serum) had similar levels as medium taken from control cells indicating that control cells were not actively secreting these proteins.

Table S2. Metabolite Concentrations in Proliferating and Senescent HCT116 cells

Metabolite	nmol metabolite/gm protein					
	Mean (Con)	sd (Con)	Mean (Sen)	sd (Sen)	ratio (Sen/Con)	pvalue
**AMP**	1,065	70	419	244	0.393	0.036
**ADP**	10,094	4,085	5,599	2,878	0.495	NS
**ATP**	180,699	5,954	123,393	939	0.683	0.003
**total Adenylate**	191,857	7,993	129,411	2,214	0.727	0.015
**Adenylate Energy Charge**	0.968	0.010	0.975	0.013	1.007	NS
**GMP**	266	16	147	29	0.551	NS
**GDP**	1,999	873	1,133	556	0.567	NS
**GTP**	46,839	3,226	31,362	1,382	0.670	0.007
**total Guanylate**	49,105	4,015	32,593	812	0.664	0.016
**Guanylate Energy Charge**	0.975	0.007	0.979	0.011	1.005	NS
**Phosphocreatine**	63,229	7,367	48,780	1,767	0.771	NS
**creatine**	218,131	8,345	229,766	10,593	1.100	NS
**Phosphocreatine/creatine**	0.290	N.A.	0.212	N.A.	0.73	N.A.
**Glutathione (GSH)**	567,379	48,943	347,769	6,803	0.613	0.015
**Glutathione (GSSG)**	67,921	37,946	23,976	1,588	0.353	NS
**Total Glutathione**	703,220	26,965	395,720	4,727	0.563	0.002
**GSH/GSSG ratio**	10.154	4.835	14.556	1.164	1.433	NS
**NADPH**	608	88	305	103	0.502	0.019
**NADP+**	984	139	814	199	0.827	NS
**NADPH/NADP+ ratio**	0.622	0.095	0.407	0.198	0.655	NS
**NADH**	1,727	287	2,040	45	1.182	NS
**NAD+**	22,041	1,985	19,518	1,051	0.886	NS
**NADH/NAD+**	0.078	0.006	0.105	0.004	1.343	0.005
**Phosphoenolpyruvic acid**	304	55	50	5	0.164	0.015
**3-Phosphoglyceric acid**	1,668	167	485	147	0.291	0.001
**2,3-Diphosphoglyceric acid**	280	N.A.	44	5	0.157	N.A.
**2-Phosphoglyceric acid**	171	22	54	23	0.313	0.003
**Dihydroxyacetone phosphate**	3,738	2,041	875	239	0.234	NS
**Fructose 1,6-diphosphate**	7,841	1,983	3,860	609	0.492	NS
**Fructose 6-phosphate**	465	84	396	37	0.852	NS
**Fructose 1-phosphate**	976	150	1,633	340	1.674	NS
**Glucose 6-phosphate**	2,079	342	1,729	52	0.831	NS
**Glucose 1-phosphate**	190	49	179	87	0.941	NS
**UDP-glucose**	10,759	218	10,780	550	1.002	NS
**Lactic acid**	314,791	31,783	642,905	49,994	2.042	0.001
**Malic acid**	40,308	3,843	23,510	379	0.583	0.016
**Fumaric acid**	7,480	710	3,827	453	0.512	0.003

Table S2 Metabolite Concentrations in Proliferating and Senescent HCT116 cells

**Succinic acid**	**3,535**	**735**	**1,117**	**160**	**0.316**	**0.025**
**2-Oxoglutaric acid**	11,319	2,181	9,622	823	0.850	NS
**Isocitric acid**	894	188	1,810	415	2.024	0.045
**cis-Aconitic acid**	412	50	957	37	2.322	<0.001
**Citric acid**	26,539	3,172	47,287	2,846	2.289	0.002
**Pyruvic acid**	3,853	369	12,685	880	3.292	0.001
**Coenzyme A**	997	217	993	88	0.996	NS
**6-phosphoglucoronic acid**	670	76	375	227	0.6	0.1
**Ribulose 5-phosphate**	148	222	19	23	0.131	NS
**Ribose 1-phosphate**	186	19	60	16	0.325	0.001
**Ribose 5-phosphate**	148	222	19	23	0.131	NS
**PRPP**	1,552	269	250	134	0.161	0.005
**Glutamine**	835,869	24,846	123,183	15,442	0.147	<0.001
**Serine**	135,454	5,724	22,262	832	0.164	0.001
**Methionine**	69,023	3,025	20,849	213	0.302	0.001
**Histdine**	55,560	1,940	16,830	341	0.303	0.001
**Tyrosine**	128,910	4,396	40,337	684	0.313	0.001
**Tryptophan**	23,874	903	7,698	169	0.322	0.001
**Isoleucine**	193,489	6,330	63,596	784	0.329	0.001
**Leucine**	192,974	5,912	63,921	2,024	0.331	<0.001
**Phenylalanine**	126,237	3,529	42,429	499	0.336	<0.001
**Valine**	196,806	4,158	73,645	414	0.374	<0.001
**Glutamic acid**	1,035,628	18,784	411,642	11,381	0.397	<0.001
**Arginine**	12,313	610	5,603	189	0.455	0.001
**Aspartic acid**	56,680	1,799	27,245	834	0.481	<0.001
**Lysine**	38,086	1,515	21,007	222	0.552	0.002
**Threonine**	628,246	25,517	405,872	4,832	0.646	0.003
**Glycine**	746,033	33,526	509,412	5,665	0.683	0.006
**Cysteine**	780	130	559	N.A.	0.718	N.A.
**Asparagine**	64,026	2,859	48,163	973	0.752	0.006
**Alanine**	317,677	13,377	477,591	13,017	1.503	<0.001
**Proline**	N.A.	N.A.	192,390	9,354	1<	N.A.
**Spermine**	894	171	2,078	537	2.323	0.051
**Spermidine**	1,460	106	1,409	250	0.965	NS
**Putrescine**	2,212	180	298	41	0.135	0.002
**Ornithine**	1,871	37	743	100	0.397	0.001
**5-Adenosylmethionine**	1,691	875	816	51	0.483	NS
**Citruline**	2,142	55	464	34	0.217	<0.001
**Putrescine/Spermidine**	1.517	0.106	0.213	0.009	0.140	0.002
